# Cell Survival Following Radiation Exposure Requires miR-525-3p Mediated Suppression of ARRB1 and TXN1

**DOI:** 10.1371/journal.pone.0077484

**Published:** 2013-10-16

**Authors:** Anne Kraemer, Zarko Barjaktarovic, Hakan Sarioglu, Klaudia Winkler, Friederike Eckardt-Schupp, Soile Tapio, Michael J. Atkinson, Simone Moertl

**Affiliations:** 1 Helmholtz Center Munich, German Research Center for Environmental Health, Institute of Radiation Biology, Neuherberg, Germany; 2 Helmholtz Center Munich, German Research Center for Environmental Health, Department of Protein Science, Proteomics Core Facility, Neuherberg, Germany; 3 Chair of Radiation Biology, Technical University Munich, Munich, Germany; University of Kentucky College of Medicine, United States of America

## Abstract

**Background:**

microRNAs (miRNAs) are non-coding RNAs that alter the stability and translation efficiency of messenger RNAs. Ionizing radiation (IR) induces rapid and selective changes in miRNA expression. Depletion of the miRNA processing enzymes Dicer or Ago2 reduces the capacity of cells to survive radiation exposure. Elucidation of critical radiation-regulated miRNAs and their target proteins offers a promising approach to identify new targets to increase the therapeutic effectiveness of the radiation treatment of cancer.

**Principal Findings:**

Expression of miR-525-3p is rapidly up-regulated in response to radiation. Manipulation of miR-525-3p expression in irradiated cells confirmed that this miRNA mediates the radiosensitivity of a variety of non-transformed (RPE, HUVEC) and tumor-derived cell lines (HeLa, U2-Os, EA.hy926) cell lines. Thus, anti-miR-525-3p mediated inhibition of the increase in miR-525-3p elevated radiosensitivity, while overexpression of precursor miR-525-3p conferred radioresistance. Using a proteomic approach we identified 21 radiation-regulated proteins, of which 14 were found to be candidate targets for miR-525-3p-mediated repression. Luciferase reporter assays confirmed that nine of these were indeed direct targets of miR-525-3p repression. Individual analysis of these direct targets by RNAi-mediated knockdown established that ARRB1, TXN1 and HSPA9 are essential miR-525-3p-dependent regulators of radiation sensitivity.

**Conclusion:**

The transient up-regulation of miR-525-3p, and the resultant repression of its direct targets ARRB1, TXN1 and HSPA9, is required for cell survival following irradiation. The conserved function of miR-525-3p across several cell types makes this microRNA pathway a promising target for modifying the efficacy of radiotherapy.

## Introduction

MicroRNAs (miRNAs) are short, highly conserved, non-coding RNA molecules that selectively regulate protein production through translational repression and cleavage of target mRNAs [1-3]. Data from the ENCODE genome project suggest that more than 1000 miRNA transcription units are present in the human genome; yielding an even greater number of miRNAs through RNA editing [4]. Each miRNA species has the potential to regulate more than 100 different mRNA targets, and it has been suggested that the expression of approximately 60% [5] of all protein-coding genes is controlled by miRNAs [6,7]. Multiple stress response pathways, such as cell death [8-10], DNA damage processing [11] and drug sensitivity [12], may be regulated by miRNAs.

Changes occur in miRNA expression after irradiation of normal human cells [13-15], cancer cell lines [16,17], tumor samples [18] as well as in mice [19]. Comparisons between these studies reveal a large compendium of radiation-regulated miRNAs, with surprisingly little overlap between different tissues. This suggests that the set of radiation responsive miRNAs is highly specific for cell type, radiation dose and time point [20]. Modulation of specific miRNAs reveals they can have both pro- and anti-survival functions following exposure to radiation. Wu et al. found that miR-148b expression was increased after radiation and enhanced the radiosensitivity of Non-Hodgkin Lymphoma cells by promoting apoptosis [21]. Similarly, the overexpression of let-7a decreased K-Ras expression and radiosensitized lung cancer cells [22], whilst increased miR-521 expression sensitized prostate cancer cells to radiation treatment through the regulation of the DNA repair protein CSA [16]. On the other hand, silencing of miR-21 increased radiosensitivity through inhibition of the PI3K/AKT pathway and autophagy in malignant glioma cells [23]. A radio-protective role was also shown for miR-125a and miR-189 in primary endothelial cells; their inhibition lead to a reduction in clonogenic survival [15]. 

Endothelial cells are highly sensitive to ionizing radiation [24,25], and damage to the normal tissue vasculature due to endothelial cell killing is a factor in limiting the doses that may be applied in radiation therapy. We have previously investigated miRNA expression changes during the radiation response of endothelial cells [13]. We have shown that inhibition of the transient increase in miR-525-3p expression that follows exposure to radiation reduced cellular survival by increasing apoptosis in both the endothelial cell line EA.hy926 and primary endothelial HUVEC cells. Several predicted miR-525-3p target mRNAs have functions that may be critical to the radiation response [13]. However, it is necessary to validate such candidate miRNA targets experimentally in order to understand the function of the miRNA regulated networks in the radiation response [26,27]. 

We now show that miR-525-3p is involved in the radiation response of several different cell types. Using a global proteome profiling approach we have identified 21 candidate proteins that are regulated by miR-525-3p after radiation. Of these, we determined that 9 were direct targets of miR-525-3p translational repression. Subsequent analysis identified the miR-525-3p targets arrestin beta 1 (ARRB1), thioredoxin (TXN1) and 70 kDa heat shock protein 9 (HSPA9) to be essential regulators of cellular radiation sensitivity.

## Material and Methods

### Cell culture, transfection and irradiation

The human endothelial-like cell line EA.hy926 [28] was maintained in Dulbecco’s Modified Eagle´s Medium (D-MEM medium, PAA Laboratories, Austria) supplemented with 10% fetal calf serum (FCS), 5 mM hypoxanthine, 20 μM 4-aminopteroic acid, and 0.8 mM thymidine (HAT selection supplements, PAA Laboratories, Austria). The human cervical cancer cell line HeLa was maintained in RPMI medium 1640 (PAA Laboratories, Austria) supplemented with 10% FCS [29]. The hTERT1-immortalized human RPE cell line (Clontech Laboratories, France) was grown in D-MEM / F12 medium (Gibco BRL Life Technologies, Germany) containing 2.5 mM L-glutamine, 10% FCS, 0.25% sodium bicarbonate [30]. The human osteosarcoma cell line U2-OS (HTB-96, American Type Culture Collection (ATCC)) was grown in D-MEM medium (Invitrogen, Germany) supplemented with 2% L-glutamine (Invitrogen, Germany) and 10% FCS. All cell lines were grown at 37 °C in a humified atmosphere of 5% CO_2_. Mycoplasma contamination was ruled out by routine control testing using a luminescence-based detection kit (MycoAlert, Lonza, USA). 

For transfection with small RNA molecules 2 x 10^5^ cells were seeded onto 60 mm culture plates containing 3 ml D-MEM with 10% FCS and grown to 50-70% confluence. Twenty-four hours later these cells were transfected with either miRNA inhibitor (100 pmol anti-miR-525-3p, Exiqon, Denmark), a non-specific scrambled miRNA (100 pmol anti-miR-control, Exiqon; AllStar negative control, Qiagen, Germany), precursor(pre)-miR-525-3p (100 pmol pre-miR-525-3p, Exiqon, Denmark) or specific siRNA oligonucleotides (Qiagen, Germany) using Lipofecatmine™ RNAiMAX transfection reagent (Life Technologies, Germany) according to the manufacturer’s instructions. Ionizing radiation was delivered to exponentially growing cells at the indicated doses using a Caesium-137 gamma source (HWM-D 2000, Waelischmüller, Germany) operated at a dose rate of 0.49 Gy/min.

### Analysis of miRNA expression

For the quantification of miR-525-3p expression total cellular RNA was extracted 0 h, 2 h, 4 h, 6 h, 24 h and 48 h after irradiation using the mirVana™ miRNA Isolation Kit (Ambion Inc., USA). The quality and concentration of RNA was determined with an Infinite200 NanoQuant (TECAN, Switzerland). Hsa-miR-525-3p expression was quantified using the TaqMan Single MicroRNA Assay (Applied Biosystems, USA) according to the manufacturer’s instructions. The level of miRNA was calculated following the 2^-ΔΔCt^ method using the small nucleolar housekeeping RNA (snoRNA) RNU44 as the internal reference. 

### Proteomic analysis

To identify miRNA-regulated proteins EA.hy926 cells were harvested by trypsinisation 12 h after irradiation in the presence of either anti-miR-525-3p or a non-specific scrambled miRNA. Two-dimensional gel-electrophoresis (2D-DIGE) analysis was performed with three biological replicates for each treatment. Cells were lysed in 4% SDS, 100 mM Tris HCl pH 7.6, 100 mM (0.1 M) DTT supplemented with EDTA-free protease and phosphatase inhibitor cocktails (Roche Diagnostics, Germany). The soluble proteins were precipitated with the 2D Clean-Up Kit (GE Healthcare, Germany) and the protein concentration was measured by the Bradford assay using bovine serum albumin (Sigma-Aldrich, Germany) as standard. Protein lysates were labeled with the cyanine dyes (Cy3, Cy5 and Cy2; CyDye^TM^ DIGE Fluor minimal dyes, GE Healthcare, Germany) according to the manufacturer’s instructions. Rehydration of immobilized pH gradient strips (24 cm; pH 3–11 nonlinear range; GE Healthcare, Germany) was performed with a mixture of the Cy-labeled samples in the dark at room temperature for 16 h. Isoelectric focusing was performed using immobilized pH gradients on an IPGphor3 apparatus (GE Healthcare; Germany) with the following conditions: 12 h passive rehydration, rapid 300 V for 3 h, gradients from 300 to 1000 V for 4 h, 1000 to 3500 V for 2 h 30 min, 3500 to 10000 V for 3 h 30 min and finally rapid 10000 V for 5 h corresponding a total voltage of 82 kVh. Equilibration and running of the 12% polyacrylamide gel was performed as described previously [31]. 

Identification of deregulated proteins was done by the computer program Decyder as previously described [32,33]. Protein spots were considered to be differentially regulated if a statistically significant difference in intensity was achieved at the 95% confidence level, and if the standardized average spot volume ratio exceeded 1.3-fold and p ≤ 0.05. 

Destaining of the silver stained 2D spots and in-gel trypsin digestion was performed prior to mass spectroscopy, as described previously [31]. Mass spectra of abundant protein spots were acquired using MALDI-TOF/TOF mass spectrometry (Proteomics Analyzer 4700, Applied Biosystems, USA). For less abundant spots the identification was made by LC-MS/MS linear quadrupole ion trap-Orbitrap mass spectrometry (LTQ Orbitrap XL, Thermo Fisher, Germany). The configurations and the experimental set up of both machines were as described previously [31]. The GPS Explorer ™ Software (version 3.6, Applied Biosystems, USA) was used for MALDI-TOF/TOF spectra analyses. Scaffold (version 3_00_07, Proteome Software Inc., USA) was used to validate MS/MS-based peptide and protein identifications obtained by LC-MS/MS. Carbamidomethylation was set as the fixed modification and oxidized methionine as the variable modification. The acquired MS/MS spectra were searched with Protein pilot software 3.0 against the Swiss-Prot database (updated August 2010; 519348 sequences, 183273162 residues) using Mascot 2.3.02 with the following parameters: As taxon we chose human and as enzyme trypsin allowing up to one missed cleavage. Peptide identifications were accepted if they were established with a greater than 80 % probability as specified by the Peptide Prophet algorithm. Proteins were identified if they showed a greater than 95.0% probability and contained at least 2 unique identified peptides. 

### Immunoblot analysis

Immunoblotting was performed for the validation of deregulation of selected proteins. EA.hy926 cells were lysed in RIPA buffer (150 mM NaCl, 10 mM Tris-HCl, pH 7.2, 0.1% SDS, 1% Triton X-100, 1% Na-desoxycholate, 5 mM EDTA) supplemented with 1 mM protease inhibitors (sodium orthovanadate and phenylmethanesulfonyl fluoride) (Roche, Germany) for 20 min on ice. Western blot analysis was accomplished according to standard procedures using enhanced chemiluminescence detection (Amersham, Germany). For detection of the proteins primary antibodies directed against beta-arrestin-1 (#15361-1-AP, Proteintech, USA), thioredoxin 1 (#2285, Cell Signaling, USA), heterogeneous nuclear ribonucleoprotein K (#4675, Cell Signaling, USA) and the 70kDa heat shock protein 9 (#2816, Cell Signaling, USA) were used. Protein loading was monitored by the detection of actin (#sc1616, Santa Cruz, USA) or PCNA (#sc25280, Santa Cruz, USA). HRP (horse radish peroxidase)-conjugated anti-mouse, anti-goat or anti-rabbit antibodies (Santa Cruz, USA) were used to reveal binding of primary antibodies. Quantification of digitized images was performed using TotalLabTL 100 software (TotalLab, UK). 

### In silico identification of potential miR-525-3p target sequences

The FUZZNUC program (European Molecular Biology Open Software Suite, EMBOSS, www.emboss.org) [34] was used to search for complete complementarities to the 8mer, 7mer-A1 and 7mer-m8 seed sequences of miR-525-3p. Putative target genes were predicted using five different software tools, namely TargetScan (www.targetscan.org), RNA22 (http://cbcsrv.watson.ibm.com/rna22.html), MicroCosm (http://www.ebi.ac.uk/enright-srv/microcosm/htdocs/targets/v5/), miRWalk (http://www.umm.uni-heidelberg.de/apps/zmf/mirwalk/) and DIANA-microT (http://diana.cslab.ece.ntua.gr/microT/). 

### Luciferase reporter assay to identify mRNAs directly targeted by miR-525-3p

Candidate gene cDNA sequences were obtained by PCR amplification of reverse transcribed EA.hy926 cell mRNA using the primer sets indicated in Table 1. These PCR fragments were directly cloned into the pmirGLO Dual-Luciferase miRNA Target Expression Vector (Promega, USA) using the PmeI and SbfI restriction sites. The vector uses dual-luciferase technology, with *Firefly* luciferase (luc2) being the reporter used to quantify miRNA regulation of translation and *Renilla* luciferase (hRluc-neo) being the non-regulated internal control. The identity and integrity of all constructs were confirmed by DNA sequencing. 

Transfection of reporter constructs into endothelial EA.hy926 cells was performed using Lipofectamine 2000 (Invitrogen, Germany) in duplicate 96-well plates. Five nmol of either pre-miR-525-3p, anti-miR-525-3p or the unspecific control oligonucleotides were transfected along with the 0.2 μg pmirGlo Dual-Luciferase construct harboring cDNA of putative miR-525-3p regulated targets. Twenty-four hours post transfection, cells were lysed with passive lysis buffer and the activities of *Firefly* luciferase and *Renilla* luciferase were measured using the dual Luciferase Assay System (Promega, USA). The ratio of *Firefly* luciferase and *Renilla* luciferase was expressed as normalized luciferase activity to compensate differences in transfection efficiencies. The relative luciferase activity was determined as the ratio between normalized luciferase activities of cells transfected with pre-miR-525-3p and control miRNA.

### Cell viability and apoptosis assay

Cell proliferation and apoptosis were examined as described previously [13]. 

### Statistical analysis

Data are presented in figures as mean ± s.e.m.. Significance of n-fold changes were calculated by using the one sample t-test. Unpaired, two-tailed t-test was used to compare two independent groups. For all statistical analysis, Prism for Windows, version 5.0 (GraphPad Software, USA) was used, and *p* < 0.05 was considered statistically significant. For the analysis of proteome data (Table 1 and 2) statistics were performed in DeCyder software using t-test. Proteins were considered significantly deregulated at 95% significance level (t-test, p<0.05) using three biological replicates. False Discovery Rate (FDR) correction was applied in the statistics.

**Table 1:** List of regulated proteins in anti-miR-525-3p transfected cells 12 h after irradiation compared to non-irradiated cells.

**spot**	**identified protein (gene)**	**accession number**	**n-fold change**(control transfected)	**n-fold change**(anti-miR transfected)
*up-regulated*				
17	Heat shock protein 70 kDa protein 9 (mortalin) (HSPA9)	P38646	unchanged	1.3*
18	Thioredoxin-dependent peroxide reductase, mitochondrial (PRDX3)	P30048	unchanged	1.3*
21	Heterogeneous nuclear ribonucleoprotein K (hnRNP K)	P61978	unchanged	1.3*
22	Heat shock 60kDa protein 1 (chaperonin) (HSPD1)	P10809	unchanged	1.4*
24	Esterase D/formylglutathione hydrolase, isoform CRA (ESD)	B3KT77	unchanged	1.4*
9	Chaperonin containing TCP1, subunit 2 (CCT2)	B5BTY7	unchanged	1.4*
15	Histidine triad nucleotide binding protein 1 (HINT1)	P49773	unchanged	1.4*
10	Arrestin, beta 1 (ARRB1)	P49407	unchanged	1.5*
29	Glutamate-cystein ligase (GCLM)	P48507	unchanged	1.5**
20	Proteasome activator complex subunit 2 (PSME2)	P61289	unchanged	1.5*
19	Proteasome (prosome, macropain) 26S subunit, non-ATPase, regulatory subunit 10 (PSMD10)	O75832	unchanged	1.6*
14	Peptidyl-prolyl cis-trans isomerase (PPIG)	A8K486	unchanged	1.7**
16	Thioredoxin-1 (TXN1)	P10599	unchanged	1.8*
28	Tumor protein, translationally-controlled 1 (TPT1)	Q5W0H4	unchanged	1.8*
*down-regulated*				
26	Heterogeneous nuclear ribonucleoprotein A2/B1 (HNRPA2B1)	P22626	unchanged	-1.3*
11	Acidic (leucine-rich) nuclear phosphoprotein 32 family, member B (ANP32B)	Q53F35	unchanged	-1.4*
12	Acidic (leucine-rich) nuclear phosphoprotein 32 family, member A (ANP32A)	P39687	unchanged	-1.4*
27	X-ray repair complementing defective repair in Chinese hamster cells 6 (XRCC6/KU70)	B1AHC9	unchanged	-1.4*
13	Histone H2A	B2R5B3	unchanged	-1.5*
23	Protein SCO1 homolog, mitochondrial precursor (SCOD1)	O75880	unchanged	-1.9*
25	Thymidylate synthase (TYMS)	A8K9A5	unchanged	-2.4*

In control transfected cells these proteins remain unchanged after irradiation. p-values indicate significant differences between irradiated and non-irradiated samples (*p<0.05, **p<0.01).

**Table 2:** List of regulated proteins in anti-miR-525-3p and in control transfected cells 12 h after irradiation compared to non-irradiated cells.

**spot**	**identified protein (gene)**	**accession number**	**n-fold change** (control transfected)	**n-fold change** (anti-miR transfected)
1	Obg-like ATPase 1 (OLA1)	Q9NTK5	1.3*	1.4**
2	Peroxiredoxin-1 (PRDX1)	Q06830	1.4**	1.6*
3	Prefoldin subunit 2 (PFDN2)	Q9UHV9	-3.1**	-3.1**
4	Destrin (DSTN)	B7Z9M9	-2.7**	-2.9**
5	Annexin A5 (ANXA5)	E7ENQ5	1.3*	1.4*
6	Ribosomal protein S3A (RPS3A)	A8K4W0	1.3*	1.6*
7	Cofilin-1 (CFL1)	B4E112	-2.2*	-2.8*
8	C-type lectin domain family 11 member A (CLEC11A)	Q9Y240	-1.5*	-1.5*

p-values indicate significant differences between irradiated and non-irradiated samples (*p<0.05, **p<0.01).

## Results

### Up-regulation of miR-525-3p is essential for the survival of multiple cell types after radiation

**Figure 1:**
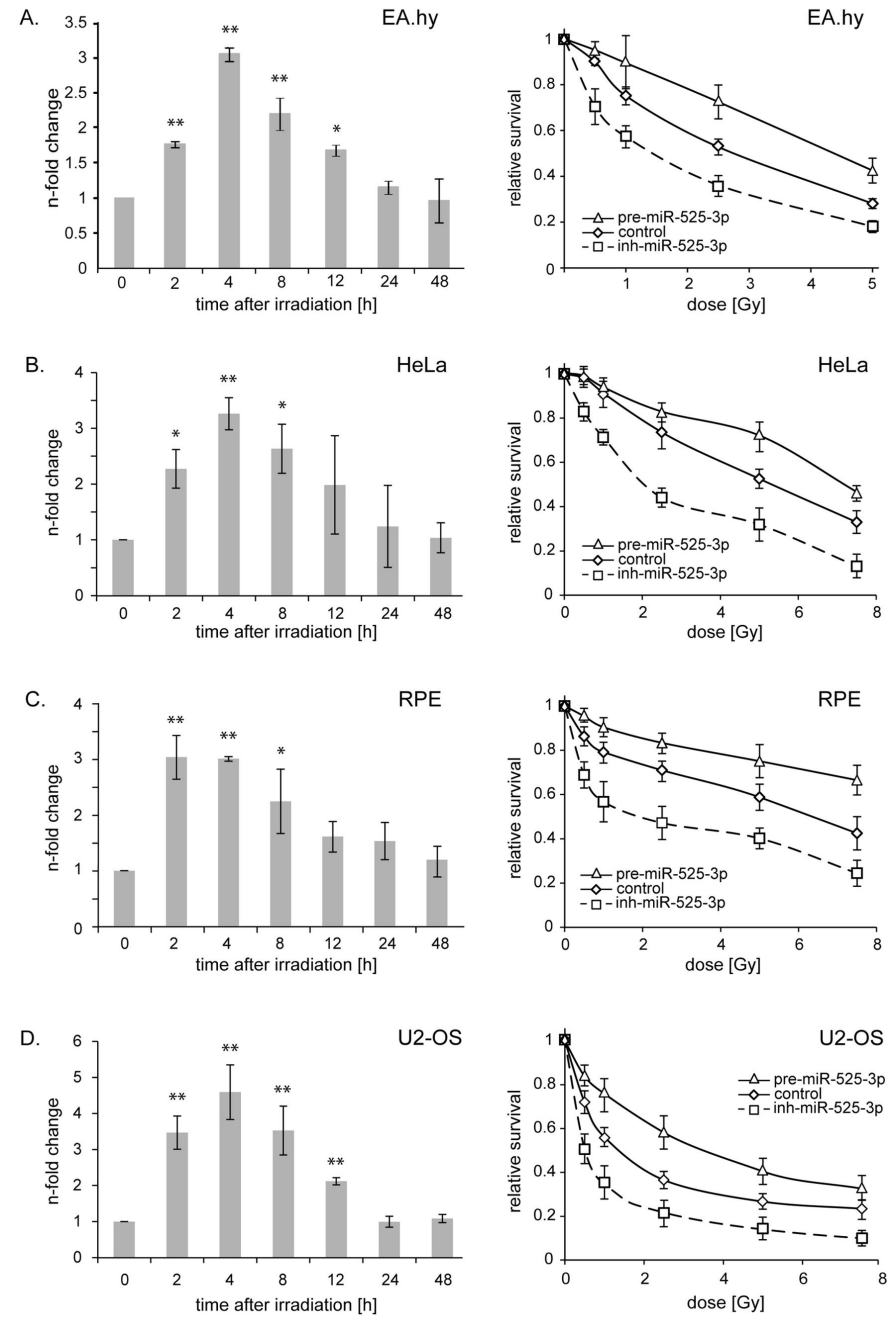
Expression of miR-525-3p is up-regulated after ionizing radiation and modulation of the miR-525-3p expression effects cell survival. (A) *left* miR-525-3p expression was examined 0, 2, 4, 8, 12, 24 and 48 h after 2.5 Gy IR in the endothelial cell line EA.hy926 by quantitative real time PCR. *right* modulation of miR-525-3p results in a change in endothelial cell proliferation after IR. Endothelial cells were transfected with pre-miR-525-3p, miR-525-3p-inhibitor or scrambled control RNA, reseeded and the cell proliferation assay was performed 5d after IR. (B) HeLa cells, (C) RPE cells, (D) U2-OS cells. The mean ± s.e.m. of three independent experiments is shown. * mark significant differences between samples harvested at the 0 h timepoint compared with the indicated time point (* p<0.05, ** p< 0.01).

The expression of miR-525-3p in EA.hy926 cells showed a rapid three-fold increase, peaking between 2 h and 4 h after 2.5 Gy irradiation. At 12 h post-irradiation the expression remained 2-fold higher than that of sham-irradiated cells, finally returning to basal levels at 24 h (Figure 1A, left). The impact of this transient up-regulation of miR-525-3p on the survival of EA.hy926 cells was studied by manipulating the cellular miR-525-3p content. Transfection of anti-miR-525-3p prior to irradiation reduced mature miR-525-3p levels in both non-irradiated and irradiated cells to less than 20% of their respective control values (Figure S1). Conversely, transfection of precursor miR-525-3p dramatically increased miR-525-3p abundance (Figure S1). The inhibition of miR-525-3p by transfection of the anti-miRNA reduced the post-radiation cell survival, while overexpression of miR-525-3p by transfection of precursor increased cell survival 5 d after irradiation (Figure 1A, right). 

Radiation-induced up-regulation of miR-525-3p was also seen in the epithelial tumor cell line HeLa (Figure 1B, left), the retinal pigment epithelium-derived cell line RPE (Figure 1C, left) and the osteosarcoma cell line U2-OS (Figure 1D, left). In each of these cell models the same effects of miR-525-3p manipulation on survival as seen in EA.hy926 cells were confirmed (Figure 1B-D, right). These results suggest that repression of the translation of miR-525-3p targets following radiation exposure contributes to the survival of multiple cell types.

### Identification of putative targets of the radiation regulated miR-525-3p

**Figure 2:**
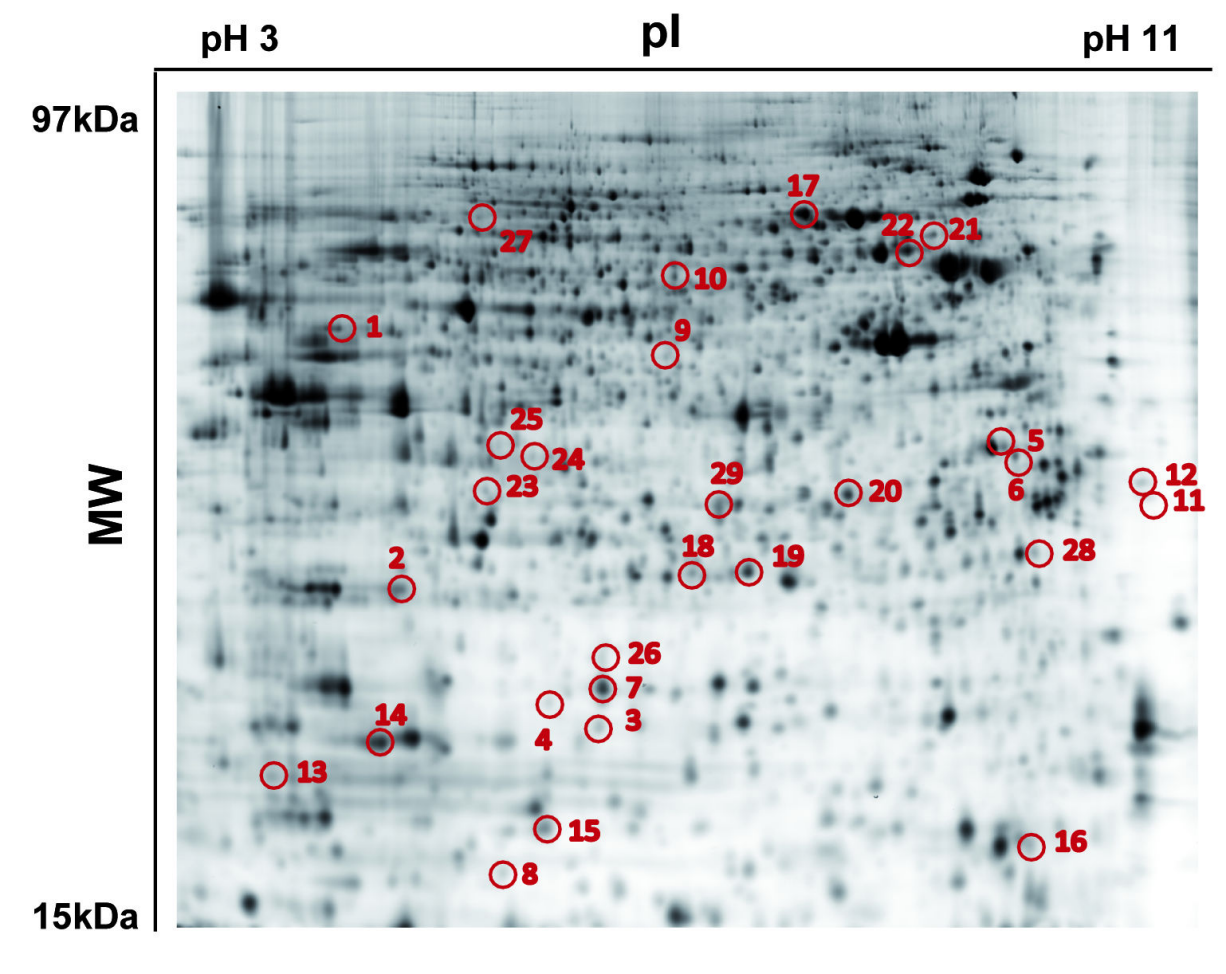
2D-DIGE gel showing the proteome of EA.hy926 cells at pH range of 3-11. Differentially regulated spots in control transfected cells (0 Gy) versus anti-miR-525-3p treated cells (2.5 Gy) are indicated with corresponding spot numbers.

The analysis of the miR-525-3p-dependent changes in the proteome of irradiated EA.hy926 cells was determined 12h after irradiation in the presence of anti-miR-525-3p (Figure 2). Fourteen candidate miR-525-3p repressed proteins were identified by 2D-DIGE (Tab 1). These proteins were all found to be increased after irradiation in anti-miR-525-3p treated cells, relative to the irradiated miR-525-3p competent (control miRNA-treated) cells. Seven additional proteins showed a down-regulation in irradiated cells in the presence of anti-miR-525-3p, suggesting an indirect regulation by the miRNA. The blockade of radiation-induced miRNA-525-3p expression had no effect on four proteins that were increased after irradiation and on a further four proteins that were decreased after radiation (Tab 2). 

**Figure 3:**
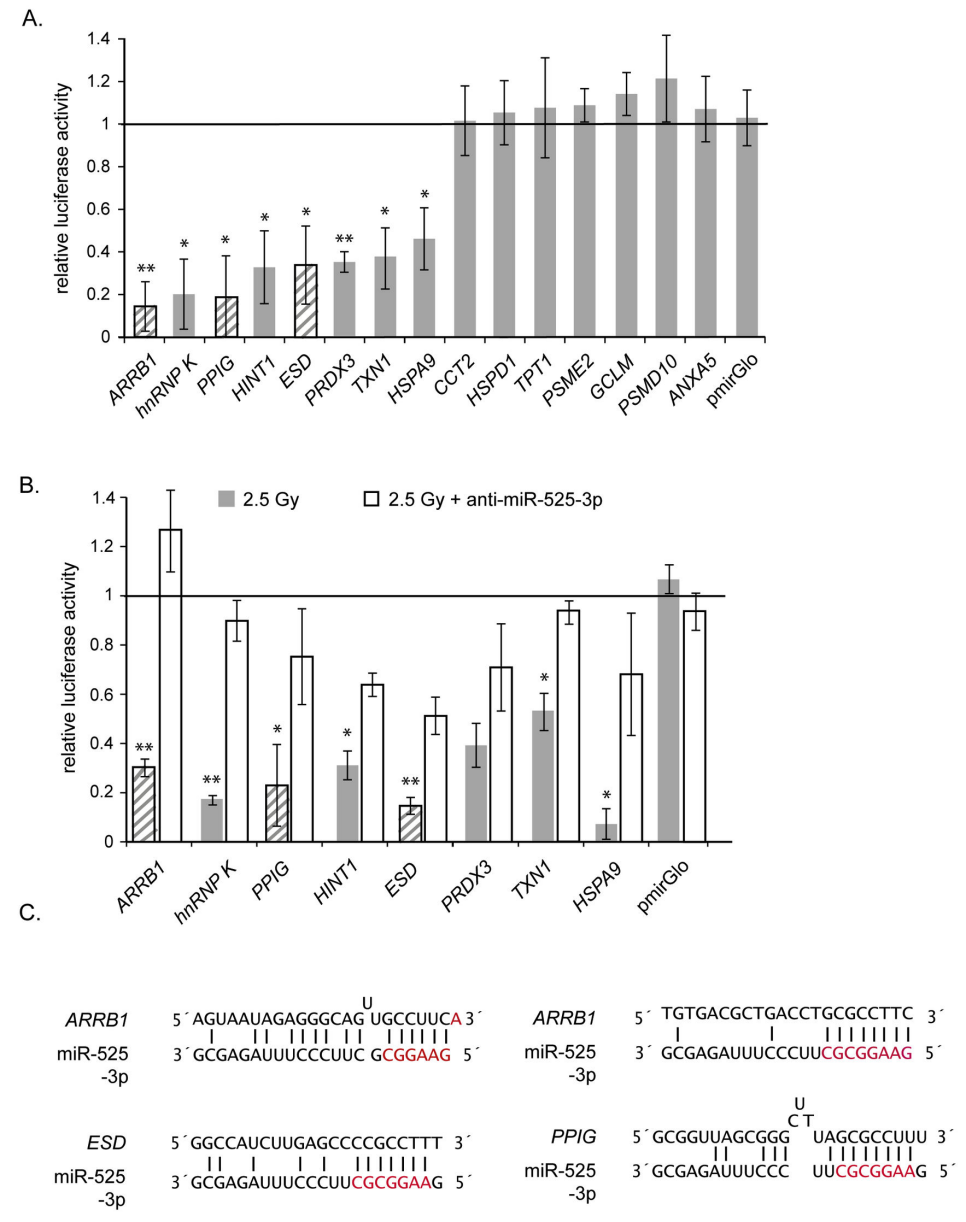
Verification of direct miR-525-3p targets using luciferase assays. (A) Relative luciferase activities after a co-transfection of luciferase constructs and control miRNA or pre-miR-525-3p in EA.hy926 cells. The mean ± s.e.m. of three independent experiments is shown. * indicate significant differences to pmiRGlo transfected cells (* p<0.05, ** p< 0.01). (B) Relative luciferase activities after co-transfection of luciferase constructs and anti-miR-525-3p or control miRNA followed by an irradiation with 2.5 Gy. The *Firefly* luciferase values were normalized for transfection with *Renilla* luciferase activity. Relative luciferase activities represent the ratio between normalized luciferase activities of pre-miR-525-3p and control miRNA transfected cells. Grey dashed bars represent sequences with perfect seed sequence matches to miR-525-3p. The mean ± s.e.m. of three independent experiments is shown. * indicate significant differences between control and anti-miR-525-3p transfected cells (* p<0.05, ** p< 0.01). (C) Complementarity of miR-525-3p sequence to the three target genes bearing perfect seed matches. The seed sequence is shown in red. Vertical lines indicate identity between miRNA sequence and corresponding gene sequence.

We performed luciferase reporter assays to establish the nature of the regulation of miR-525-3p on the expression of each of the 14 candidate repressed proteins. As miRNA-binding sites can be distributed over the whole mRNA transcript, rather than restricted to the 3´-UTR [35-37], we analyzed the complete ORF and 3´-UTR regions of each candidate transcript. The empty reporter plasmid (pmirGlo) and a reporter construct containing the cDNA sequence for a non-regulated protein (ANXA5) that lacks any putative sequence homology with miR-525-3p were used as controls. We could confirm the direct repression of reporter protein expression for 8 of the 14 candidate miR-525-3p targets in pre-miR-525-3p transfected cells (Figure 3A), establishing these as direct miR-525-3p targets. Presumably the remaining 6 proteins that did not show miR-525-3p reporter repression are repressed by miR-525-3p in a weak or indirect manner. Each of the eight reporter constructs showing repression by pre-miR-525-3p also showed down-regulation in response to an exposure to 2.5 Gy irradiation (Figure 3B). Importantly the transfection of anti-miR-525-3p was able to block the radiation-induced reduction of luciferase reporter activity for each of the 8 miR-525-3p regulated proteins (Figure 3B).

Target prediction algorithms were able to identify a stringent seed match in three of the eight directly-regulated proteins (PPIG1, ESD1 and ARRB1), with the latter containing two matches (Figure 3C). One further target (PRDX3) include a less stringent miRNA :: mRNA binding motif, whilst stringent binding regions were not predicted in the remaining four direct targets (HINT1, HSPA9, TXN1, hnRNP K) using the available homology search algorithms. Further none of the indirect targets contained stringent seed sequence matches. 

### Functional annotation of the miR-525-3p targets

To gain insights into the biological roles of the miR-525-3p target proteins in the response to radiation we performed *Gene Ontology (GO*) analysis. The differentially regulated proteins were uploaded into the UniProt knowledge database (www.uniprot.org) and the Database for Annotation, Visualization and Integrated Discovery (DAVID, http://david.abcc.ncifcrf.gov/). All 14 miR-525-3p target proteins are represented by the four GO functional annotations cell death/apoptosis, homeostasis/cell growth, post-translational modification and reduction/small metabolites (Tab 2). 

**Figure 4:**
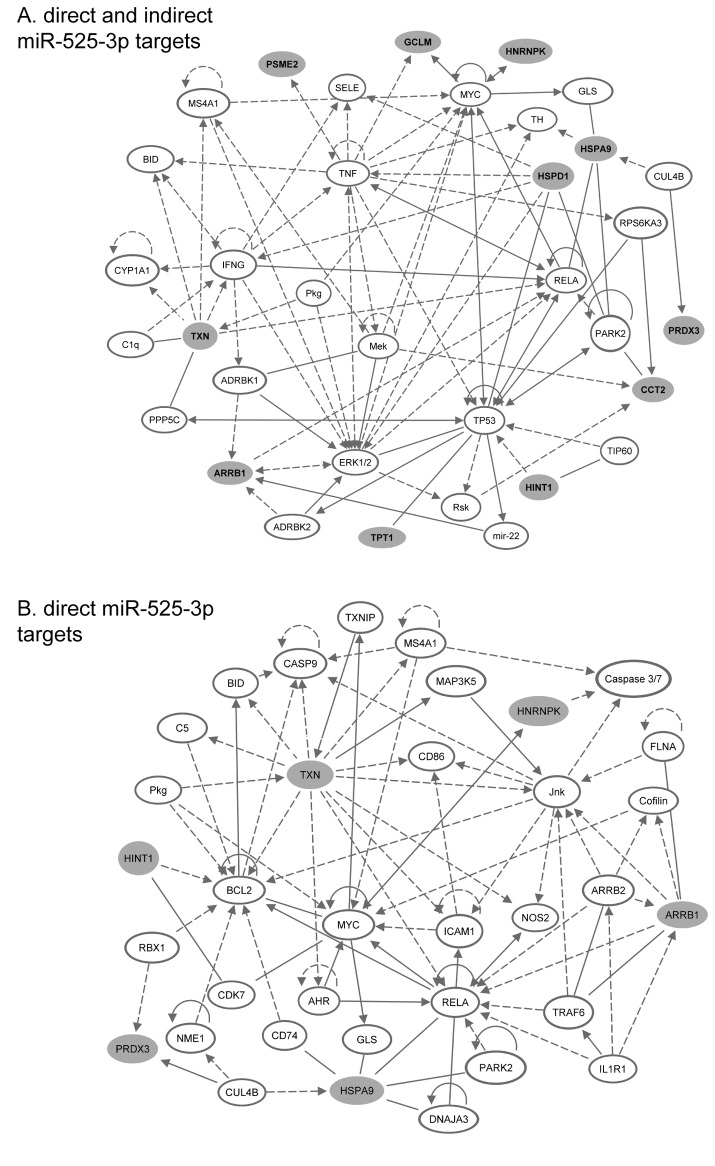
Ingenuity pathway analysis of proteins deregulated 12 h after irradiation in the absence of miR-525-3p. (A) IPA of direct and indirect miR-525-3p target proteins. The most significant network ”Cell Death and Survival, Free Radical Scavenging, Cancer” (score 26) is shown. (B) IPA of direct miR-525-3p target proteins. The most significant network “Cell Death and Survival, Organismal Injury and Abnormalities, Respiratory Disease” (score 14) is shown. Molecules in grey represent miR-525-3p target proteins. direct interaction, ------ indirect interaction.

INGENUITY Pathway Analysis (IPA) (www.ingenuity.com) was used to obtain information about potential pathways and interactions amongst the set of differentially regulated proteins [38]. The most significant IPA network was “Cell Death and Survival, Free Radical Scavenging, Cancer” with a highly significant score of 26 representing 11 of the 14 target proteins (Figure 4A). Some of the prominent nodal molecules located at the heart of this putative network are RELA (a component of the NF-κB complex), ERK1/2 (isoform 1 and 2 of extracellular signal-related kinases) and tumor suppressor protein TP53. The IPA performed on the eight direct targets identified the most significant network (score 14) as “Cell Death and Survival, Organismal Injury and Abnormalities, Respiratory Disease” and included six of the eight direct target proteins (Figure 4B). Prominent nodal molecules in this network are RELA, the transcription factor Myc and Bcl2 (B-cell lymphoma protein 2).

### The miR-525-3p direct target genes ARRB1, TXN1, HSPA9 influence radiation sensitivity

**Figure 5:**
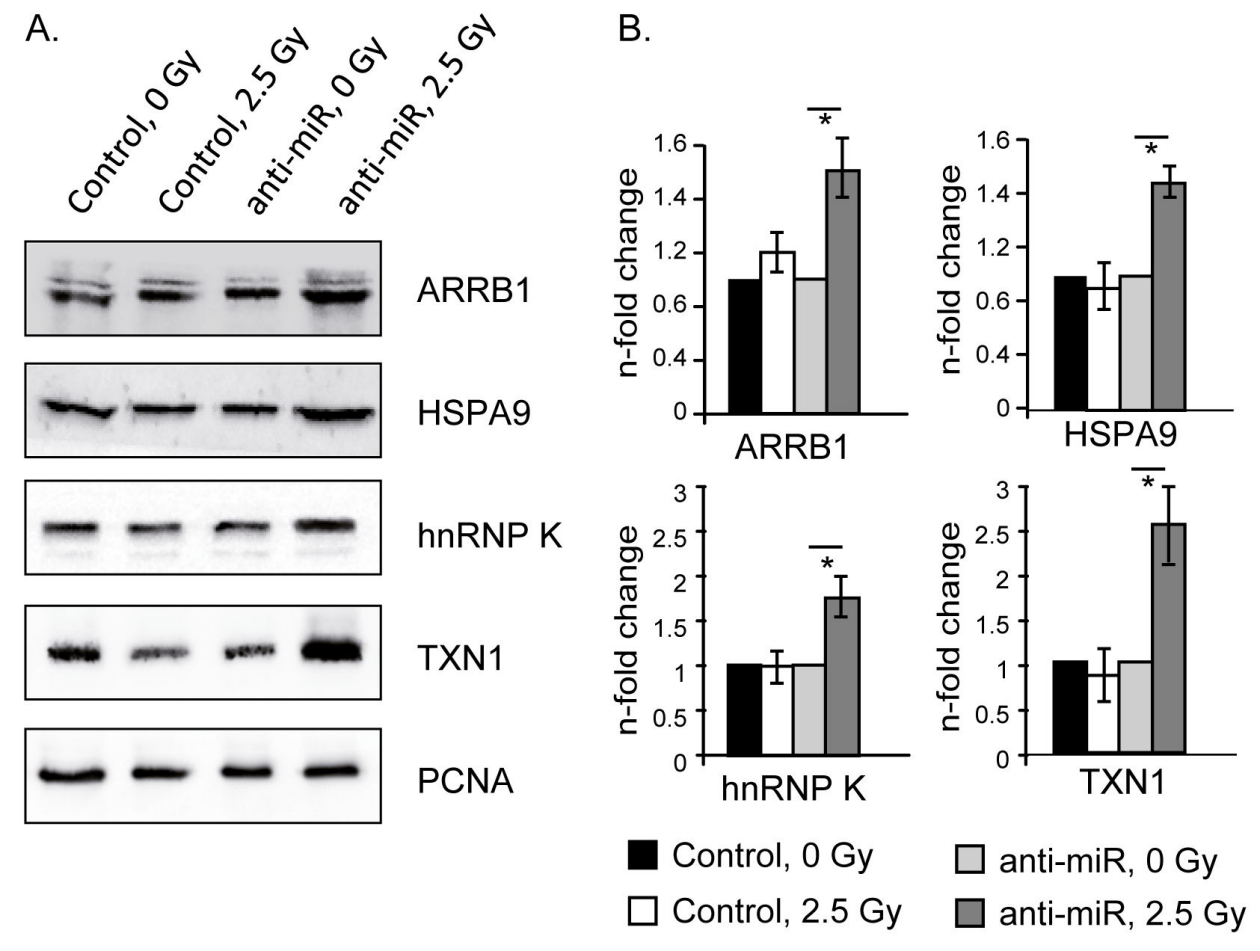
Immunoblot analysis of control and anti-miR-525-3p transfected EA.hy whole cell extracts (12 h after irradiation). (A) Representative images of the blots. (B) Fold differences between irradiated and non-irradiated samples normalized to PCNA. * indicate significant differences between irradiated and non-irradiated samples (p< 0.05). The mean ± s.e.m. of three independent experiments is shown.

Four of the directly regulated proteins (ARRB1, TXN1, HSPA9 and hnRNP K) have possible involvement in the radiation response [39-44]. Immunoblotting confirmed the results of the original proteomic screening by showing radiation-induced up-regulation of ARRB1, TXN1, HSPA9 and hnRNP K 12 h after 2.5 Gy exposures in the presence of anti-miR-525-3p (Figure 5). In the absence of anti-miR-525-3p each of these proteins remained unchanged after irradiation, confirming that the radiation-induced increase in expression of miR-525-3p is necessary to prevent increases in these proteins during the radiation response.

**Figure 6:**
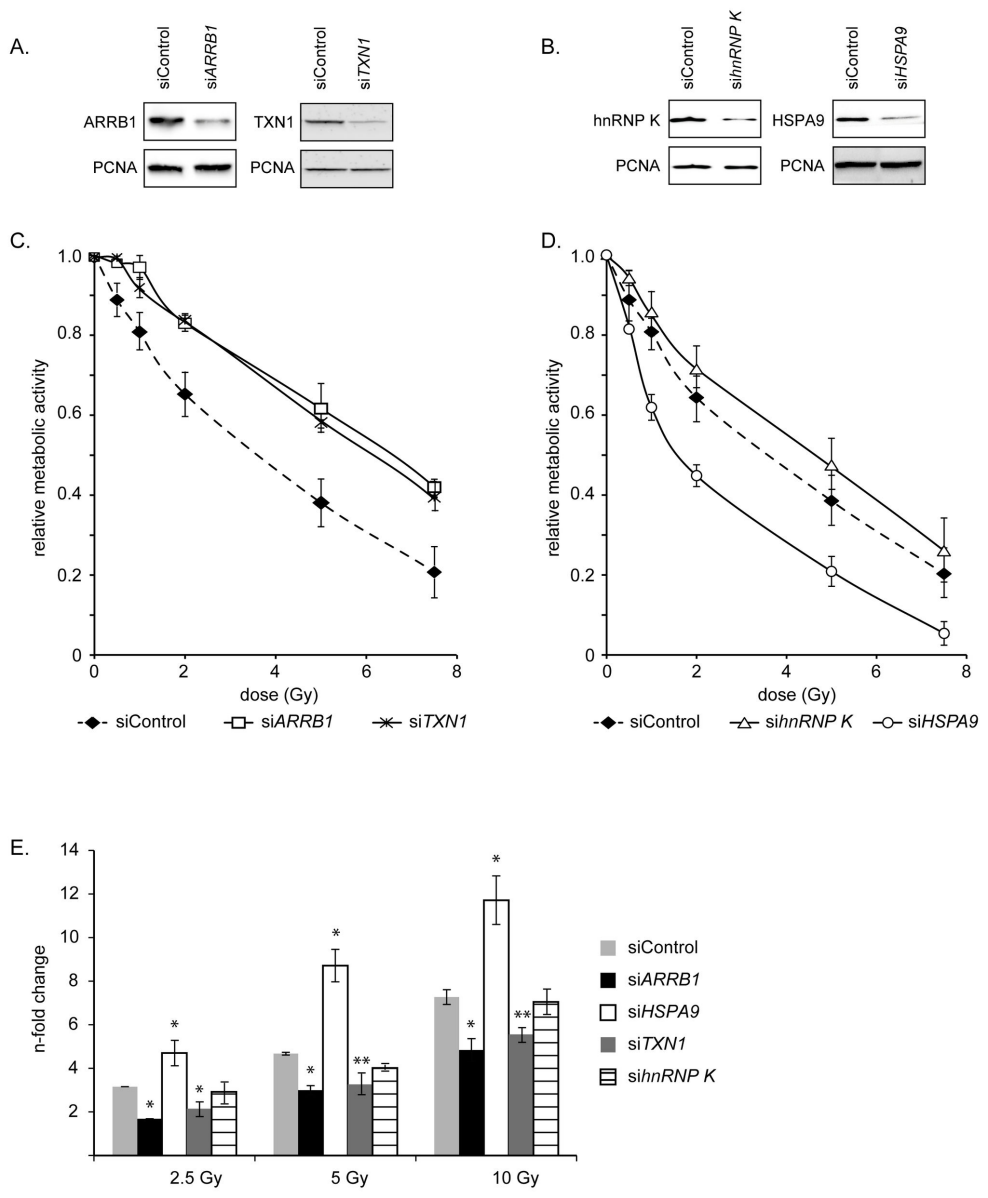
Radiation response after depletion of ARRB1, TXN1, hnRNP **K and HSPA9**. (**A**) siRNA-mediated knockdown of ARRB1 and TXN1. EA.hy926 cells were transfected with siARRB1 or with an unspecific control (siControl). ARRB1 and TXN1 were quantified 24 h after transfection by western blot. (B) siRNA-mediated knockdown of hnRNP K and HSPA9. (C) Proliferation activity after IR in ARRB1 and TXN1 knockdown cells. Depletion of ARRB1 and TXN1 results in increased radiation resistance after irradiation up to 7.5 Gy. Endothelial EAhy926 cells were transfected with siARRB1, siTXN1 or scrambled control RNA (siControl), reseeded and the cell proliferation assay was performed 5d after ionizing radiation. The mean ± s.e.m. of two independent experiments is shown. (D) Proliferation activity after IR in hnRNP K and HSPA9 knockdown cells. Depletion of hnRNP K did not change the proliferative activity and depletion of HSPA9 led to decreased proliferative activity. The mean ± s.e.m. of two independent experiments is shown. (E) Apoptosis induction in knockdown cells after IR. Apoptosis induction was quantified by sub-G1 analysis 48 h after IR. Depletion of ARRB1 and TXN1 led to decreased apoptosis, while depletion of HSPA9 increased apoptosis. * indicate significant difference to the respective siControl transfected cells (** p < 0.05, * p < 0.01). The mean ± s.e.m. of three independent experiments is shown.

To test whether ARRB1, HSPA9, TXN1 and hnRNP K actually influence cellular radiation sensitivity each of these proteins was down-regulated by RNA interference (Figure 6A). Knockdown of ARRB1 and TXN1 increased survival of EA.hy926 cells after 2.5 Gy compared to irradiated scrambled siRNA-transfected control cells (Figure 6C). Unexpectedly, the knockdown of HSPA9 decreased cellular survival, whilst depletion of hnRNP K had no impact (Fig 6BD). Quantification of sub G1 apoptotic cell numbers demonstrated a significant reduction of apoptosis after irradiation in ARRB1- and TXN1-knockdown cells. Depletion of HSPA9 increased the level of radiation-induced apoptosis, while reduction of hnRNP K had no impact (Figure 6E). Taken together, these results demonstrate that cell survival after irradiation is dependent on the coordinated miR-525-3p-mediated translational repression of both anti-survival (ARRB1 and TXN1) and pro-survival (HSPA9) targets.

## Discussion

Ionizing radiation induces changes in miRNA expression in a range of cell types. The subsequent cellular responses can often be reiterated through the manipulation of a single miRNA species. However, the mechanisms by which the miRNA changes modulate radiation sensitivity remain largely unknown with an almost complete lack of evidence identifying the protein targets that are actually regulated by radiation-responsive miRNAs [15,21-23].

Recently, we demonstrated that a radiation-induced increase in miR-525-3p is sufficient to limit the extent of cell death and apoptosis in human endothelial cells [13]. In the current study we now show that radiation-induced up-regulation of miR-525-3p occurs in a variety of other human cell lines, where it is essential for sustaining cell survival. The consistency of this function across multiple cell types suggests a conserved and important role of miR-525-3p in regulating the radiation response. This is in sharp contrast to the more restricted, cell type specific, roles suggested for other radiation-regulated miRNAs [8,45,46]. 

Our proteomic analysis identified 14 proteins that were repressed by the radiation-induced increase in miR-525-3p. The overall changes in protein expression (from -2.4 to 1.8) were subtle, in accordance with the suggested role of miRNAs as fine tuners of protein abundance [47]. Gene ontology annotation assigned the majority of the deregulated proteins to the biological process of cell death and apoptosis. This is in accordance with previous data describing radiation-induced apoptosis as one of the most important radiation-response pathways in endothelial cells [25,48,49]. It is also in agreement to our earlier experimental data showing an impaired increase in apoptosis induction in cells where the radiation-induced increase in miR-525-3p was blocked [13]. A number of the most important nodal molecules predicted by Ingenuity Pathway Analysis of the deregulated proteins are implicated in radiation-induced apoptosis. For example RELA, encodes the p65 protein that is a component of the NFκB complex activated by radiation to influence apoptosis and DNA repair [50]. The nodal molecule p53 is stabilized after radiation exposure and can induce the expression of multiple genes involved in apoptosis [51,52]. ERK-mediated signals and Bcl-2 both inhibit radiation-induced changes in the mitochondrial membrane and the subsequent cell death in lymphocytic leukemia cells [53]. Bcl-2 is an important protein in apoptosis [54] whose suppression renders cells more susceptible to radiation-induced apoptosis [55]. The nodal molecule MYC has a role in sensitizing cells to apoptosis, with inhibition of MYC by antisense oligonucleotides reducing radiation-induced apoptosis in a small-cell lung cancer cell line [56].

Eight of the 14 proteins predicted to be repressed by the radiation up-regulation of miR-525-3p were confirmed by luciferase reporter assays to be direct targets. In the absence of miR-525-3p these 8 reporter constructs were all overexpressed in irradiated cells confirming that the miR-525-3p :: target interactions occur under physiological conditions. miRNA target interaction is mainly based on a stringent base pairing between the miRNA seed sequence and the target mRNA [57]. Three of the direct targets in this study contained such stringent seed sequence matches (ESD, PPIG, ARRB1). The remaining five direct targets showed only weak predicted seed sequence interactions (HINT1, HSPA9, TXN1, hnRNP K, PRDX3). Such experimentally verified targets with poor seed sequences matches are not unusual [37,58]. It is suggested that additional 3´- pairing and pairing in centered regions of miRNAs could compensate for weaker seed sequence binding [59]. Also, a recently discovered alternative binding mechanism involving a multistep binding process with induced conformational changes in the miRNA :: mRNA duplex may support binding between miRNA and targets with poor seed sequence matches [60]. 

Four of the eight direct miR-525-3p targets, ARRB1, hnRNPK, HSPA9 and TXN1 have functions in the cellular stress response. As none of these proteins were significantly increased in miR-525-3p competent cells in response to irradiation we can assume that increases in their expression levels are suppressed during the radiation response by the action of the increase in miR-525-3p. It is possible that low-level changes in their regulation may occur below the detection limit (1.3 fold change for proteins) of our proteomic analysis. Individual analysis of the changes of these four targets after irradiation confirmed that ARRB1 and TXN1 act as negative regulators of survival. Cell survival increased after irradiation when these proteins were knocked down by siRNA. In contrast, HSPA9 has a direct pro-survival function, with HSPA9-depleted cells being more radiosensitive than controls. Integrating these results with the overall effect of miR-525-3p on radiation sensitivity we suggest that the up-regulation of miR-525-3p acts to fine tune the balance between both, the negative and the positive regulators of survival. 

The repressed protein ARRB1 indirectly regulates transcription factors involved in DNA damage processing and apoptosis in chronic stress responses through binding to regulators such as IκBα and MDM2 [61]. Suppression of ARRB1 by RNA interference increases NF-κB activity in HeLa cells and, conversely, its overexpression reduces NF-κB activity [62]. Further, ARRB1 suppresses p53 levels leading to an accumulation of unrepaired DNA damage [41]. The radiation-induced increase of ARRB1 in cells with repressed miR-525-3p may serve to reduce NF-κB activity leading to increased radiosensitivity and apoptosis.

TXN1 is a cellular redox enzyme that controls the activation of a number of transcription factors participating in the radiation response [63,64]. Byun et al. have shown that increased TXN1 expression is associated with elevated radiation sensitivity through increased apoptosis and senescence [39]. We propose similar consequences for the radiation-induced up-regulation of TXN1 in miR-525-3p blocked cells. Indeed, the siRNA-mediated knockdown of TXN1 led to increased survival and reduced apoptosis after irradiation (Figure 6C). 

HSPA9 has been shown to inactivate the transcriptional and apoptotic functions of p53 [65]. Furthermore it attenuates DNA damage after radiation exposure by maintaining the expression of TOPII alpha [43]. Consistent with this function the knockdown of HSPA9 decreased survival and increased apoptosis after irradiation (Fig 6BC). 

In summary we present evidence that miR-525-3p is an important regulator of survival in normal and in tumor-derived cell lines, and that the direct targets ARRB1, HSPA9 and TXN1 each have a direct effect on survival after irradiation. Based on the fact that three different prediction algorithms predict more than 150 miR-525-3p targets (www.miRwalk.de), we propose that ARRB1, TXN1 and HSPA9 offer only the first glimpse of a network of miR-525-3p targets that impact survival. Our results are consistent with the assumption that the effect of miR-525-3p on radiation sensitivity is the result of effects on multiple targets with both pro- or anti-survival activities. More general the conserved function of miR-525-3p across several cell types makes this microRNA a promising target for therapeutic intervention in tumor radiotherapy.

## Supporting Information

Figure S1 **Cellular abundance of mature miR-525-3p after the transfection of miR-525-3p inhibitor or precursor miR-525-3p with and without IR was measured by real-time PCR.** Irradiation of 2.5 Gy leads after 12 h to an up-regulation or miR-525-3p in control-transfected cells. Inhibition of miR-525-3p decreases the expression of miR-525-3p with and without irradiation. Transfection of pre-miR-525-3p increases the expression of the miRNA. This effect can be enhanced by irradiation. . * indicate significant difference to control transfected cells (* p < 0.01). The mean ± s.e.m. of three independent experiments is shown.
(TIF)

Table S1 **Summary of primer sequences used for the design of luciferase reporter construction.**
(DOC)

Table S2 **Functional annotation of proteins differentially expressed after miR-525-3p inhibition and irradiation by GO term analysis and by Ingenuity Pathway Analysis (IPA); IPA network A (direct and indirect targets)** : “Cell Death and Survival, Free Radical Scavenging, Cancer; IPA network B (direct targets): Cell Death and Survival, Organismal Injury and Abnormalities, Respiratory Disease .
(DOCX)
