# Supplementary figures and images for: Cell Survival Following Radiation Exposure Requires miR-525-3p Mediated Suppression of ARRB1 and TXN1
Source: PLoS One. 2013 Oct 16;8(10):e77484. doi: 10.1371/journal.pone.0077484 (PMC3797807; doi:10.1371/journal.pone.0077484)

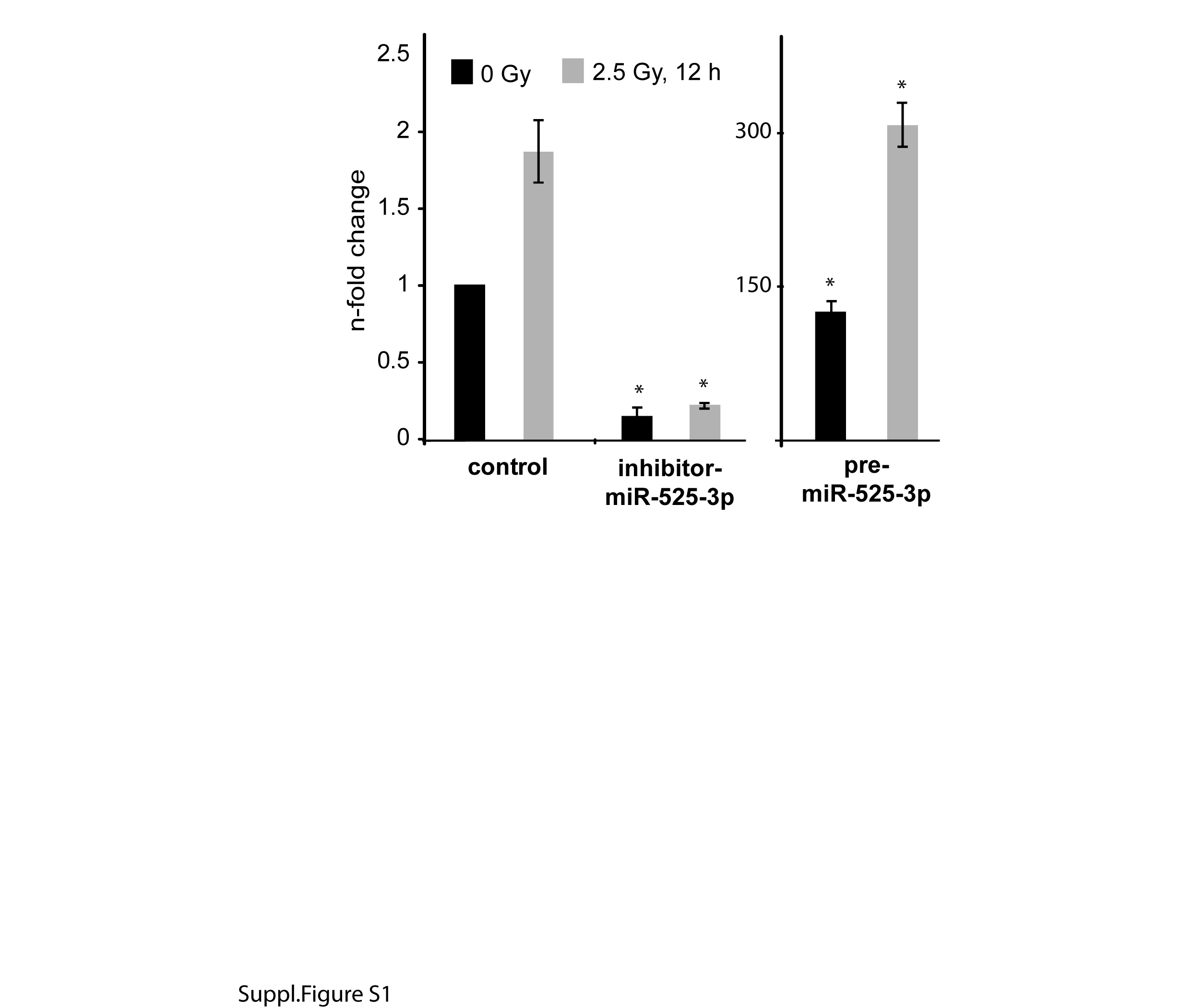

Supplement: Figure S1 — Cellular abundance of mature miR-525-3p after the transfection of miR-525-3p inhibitor or precursor miR-525-3p with and without IR was measured by real-time PCR. Irradiation of 2.5 Gy leads after 12 h to an up-regulation or miR-525-3p in control-transfected cells. Inhibition of miR-525-3p decreases the expression of miR-525-3p with and without irradiation. Transfection of pre-miR-525-3p increases the expression of the miRNA. This effect can be enhanced by irradiation. . * indicate significant difference to control transfected cells (* p < 0.01). The mean ± s.e.m. of three independent experiments is shown. (TIF) [file pone.0077484.s001.tif]
