# Supplementary material for: Cell Survival Following Radiation Exposure Requires miR-525-3p Mediated Suppression of ARRB1 and TXN1
Source: PLoS One. 2013 Oct 16;8(10):e77484. doi: 10.1371/journal.pone.0077484 (PMC3797807; doi:10.1371/journal.pone.0077484)
Supplement: Table S1 — Summary of primer sequences used for the design of luciferase reporter construction. (DOC) [file pone.0077484.s002.doc]

Suppl Table 1: Summary of primer sequences used for the design of luciferase reporter construction

| gene | NCBI Reference sequence | sequence Forward primer | Sequence reverse primer |
| --- | --- | --- | --- |
| ANXA5 | NM_001154.3 | 5´-ATGGTTTAAACCAGCGCCAGGAAATC-3´ | 5´-GTACCTGCAGGTTTCAAGGCTAAGC-3´ |
| ARRB1 | NM_004041.4 | 5´- ATG GTTTAAAC CGACCTCGTGGACC-3´ | 5´-GTA CCTGCAGG CAGAAGCTTGGAAAC-3´ |
| CCT2 | NM_001198842.1 | 5´-ATGGTTTAAACGCGTCTCCTTGTGCC-3´ | 5´-GTACCTGCAGGTTAATGCACAGTATGAG-3´ |
| ESD | NM_001984.1 | 5´-ATGGTTTAAACGGGCATCATATGGGC-3´ | 5´-GTACCTGCAGGTTCAGAGGTGATTCAAC-3´ |
| GCLM | NM_002061.2 | 5´-ATGGTTTAAACAAGGCGCTCCTGGCG-3´ | 5´-GTACCTGCAGGGATTACAGGCATGAGG-3´ |
| HINT | NM_005340.5 | 5´-ATGGTTTAAACCGCGCAAGATTAGGTG-3´ | 5´-GTACCTGCAGGTTCACATATTCCAAC-3´ |
| HNRNPK | NM_ 002140.3 | 5´-ATGGTTTAAACCGCTTCAGTTCTGC-3´ | 5´-GTACCTGCAGGTAATAAGACACTAGAGC-3´ |
| HSPA9 | NM_004134.6 | 5´-ATGGTTTAAACATCATGCTGCGGAGC-3´ | 5- GTACCTGCAGGTAGTATCTTAGGACT-3´ |
| HSPD1 | NM_002156.4 | 5´-ATGGTTTAAACCGACGACCTGTCTC-3´ | 5´-GTACCTGCAGGTGTCACATAATTGGATAC-3´ |
| PPIG | NM_004792.2 | 5´-ATGGTTTAAACGAGGCGGTTAGCGGGC-3´ | 5´-GTACCTGCAGGTTCATTAGCAAGTCTCTC-3´ |
| PRDX3 | NM_006793.2 | 5´-ATGGTTTAAACCCATGCACCTGCTGTC-3´ | 5´-GTACCTGCAGGTgttttacaatgaatacagc-3´ |
| PSME3 | NM_005789.2 | 5´-ATGGTTTAAACGCCTCGTTGCTGAAGGTGG-3´ | 5´-GTACCTGCAGGTGGAACAGTCCAAC-3´ |
| PSMD10 | NM_002814.3 | 5´-ATGGTTTAAACACAAGTAGTTGCTGGGAC-3´ | 5´-GTACCTGCAGGACAAGCTTGACATTC-3´ |
| TPT1 | NM_003295.2 | 5´-ATGGTTTAAACGCCATCATGATTATCTACC-3´ | 5´-GTACCTGCAGGCTCTTGGTAATCTCTTCCAG-3´ |
| TXN | NM_003329.3 | 5´-atggtttaaaccgggcgtgccagtttat-3´ | 5´-gtacctgcaggccaacaagagatttttgtagc-3´ |
