# Supplementary material for: Cell Survival Following Radiation Exposure Requires miR-525-3p Mediated Suppression of ARRB1 and TXN1
Source: PLoS One. 2013 Oct 16;8(10):e77484. doi: 10.1371/journal.pone.0077484 (PMC3797807; doi:10.1371/journal.pone.0077484)
Supplement: Table S2 — Functional annotation of proteins differentially expressed after miR-525-3p inhibition and irradiation by GO term analysis and by Ingenuity Pathway Analysis (IPA); IPA network A (direct and indirect targets) : “Cell Death and Survival, Free Radical Scavenging, Cancer; IPA network B (direct targets): Cell Death and Survival, Organismal Injury and Abnormalities, Respiratory Disease . (DOCX) [file pone.0077484.s003.docx]

|  | GO term analysis | | | | Network analysis | |
| --- | --- | --- | --- | --- | --- | --- |
| Gene | **Cell death**  **apoptosis** | **Homeostasis**  **Cell growth** | **Post transcriptional**  **modification** | **Reduction/**  **synthesis**  **Small metabolites** | **IPA**  **Network**  **A** | **IPA**  **Network**  **B** |
| ARRB1 | **x** |  | **x** |  | **x** | **X** |
| CCT2 | **x** |  | **x** |  | **x** |  |
| ****ESD**** |  |  |  | **x** |  |  |
| GCLM | **x** | **x** |  |  | **x** |  |
| HINT1 | **x** |  |  |  | **x** | **x** |
| hnRNP K |  | **x** |  |  | **x** | **x** |
| HSPA9 |  |  |  |  | **X** | **x** |
| HSPD1 | **x** |  | **x** |  | **x** |  |
| PSME2 |  |  |  |  | **x** |  |
| PPIG |  |  | **x** |  |  |  |
| PRDX3 | **x** |  |  |  | **X** | **X** |
| PSMD10 |  |  | **x** |  |  |  |
| TPT1 | **x** | **x** |  |  | **x** |  |
| TXN1 | **x** | **x** |  |  | **x** | **X** |

Suppl Table 2: Functional annotation of proteins differentially expressed after miR-525-3p inhibition and irradiation by GO term analysis and by Ingenuity Pathway Analysis (IPA); IPA network A (direct and indirect targets): “Cell Death and Survival, Free Radical Scavenging, Cancer; IPA network B (direct targets): Cell Death and Survival, Organismal Injury and Abnormalities, Respiratory Disease
